# Healthcare-Associated Infections Impact Mortality in Patients Admitted to the Acute Care Hospital from the Emergency Department

**DOI:** 10.3390/jcm15041483

**Published:** 2026-02-13

**Authors:** Andrea Fabbri, Ayca Begum Tascioglu, Flavio Bertini, Barbara Benazzi, Danilo Montesi

**Affiliations:** 1 Emergency Department, Local Health Agency of Romagna, Presidio Ospedaliero Morgagni-Pierantoni, Via C Forlanini 34, 47121 Forlì, Italy; bbenazzi1@gmail.com; 2 Department of Computer Science and Engineering, University of Bologna, Mura Anteo Zamboni 7, 40126 Bologna, Italy; aycabegum.tascioglu2@unibo.it (A.B.T.); danilo.montesi@unibo.it (D.M.); 3 Department of Mathematical, Physical and Computer Sciences, University of Parma, Parco Area delle Scienze 53/A, 43124 Parma, Italy; flavio.bertini@unipr.it

**Keywords:** older age, predictors, comorbidity, mortality, nosocomial infections

## Abstract

**Background/Objectives**: Prolonged stays in overcrowded emergency departments (EDs) may contribute to an increased risk of healthcare-associated infections (HAIs) and therefore mortality. Early identification of the risk profile of these patients could reduce both complications and adverse outcomes. The study aimed to verify whether the development of an HAI was associated with increased mortality. **Design, settings and participants**: This retrospective multicentre study involved all subjects who required urgent admission to an acute care hospital from the ED between 2023 and 2024. **Outcome measures:** A Cox proportional hazards model was used to test 30-day mortality. **Results:** Among the 20,234 patients considered for analysis, the mean age was 79 years (20) (median (IQR)), and a total of 1575 (7.8%) had died at 30 days. The main features selected for predicting mortality were in order of importance, diagnosis of neoplasm, older age, NEWS, diagnosis of infectious diseases, HAIs, diagnosis of respiratory diseases, CCI, priority level on arrival and male gender, yielding an accuracy of 0.804 ± 0.012. HAI occurrence was associated with a mortality risk ratio of 1.518 (95% confidence intervals (CI): 1.338–1.721; *p* < 0.001). The risk was higher for bloodstream infections (2.54; 2.12–3.06) and pneumonia (1.44; 1.20–1.73). **Conclusions**: The occurrence of HAIs was associated with an increased risk of mortality in patients admitted to acute care hospitals from the ED. This risk was particularly elevated in case of bloodstream infections and pneumonia.

## 1. Introduction

### 1.1. Background/Rationale

Healthcare-associated infections (HAIs), defined as infections occurring on or after the third day of hospital admission, are more prevalent among the elderly, the seriously ill, those with prolonged stays and those undergoing invasive procedures [1].

Patients with HAIs experience adverse events which can be caused by antibiotic resistance. This can lead to higher mortality rates and put a huge strain on healthcare systems [2,3].

Most represented HAIs categories reported by The European Centre for Disease Prevention and Control (ECDC) [4] in acute care hospitals are, in order of frequency, pneumonia (PN), urinary tract (UT) infections, surgical site (SS) infections, and bloodstream (BS) infections, followed by gastrointestinal (GI) infections and skin and soft tissue (SST) infections.

A crowded environment, such as an ED, in which many patients with different complaint severities and undiagnosed diseases coexist for long periods, can certainly increase the risk [5,6]. Identification of subjects at risk could allow for early diagnosis and treatment, thus reducing the impact of later complications and unfavourable outcomes [7].

### 1.2. Objectives

We aimed to evaluate whether developing HAIs affects mortality during hospital stays.

## 2. Materials and Methods

### 2.1. Study Design, Setting

This is a retrospective multicentre analysis of all digital health records collected from the official registry from four 1st-level EDs of the Local Health Agency of Romagna, Italy, between 1 June 2023 and 31 December 2024 (18 consecutive months) including 495,631 encounters/1,172,853 inhabitants.

### 2.2. Patients

**Figure 1:**
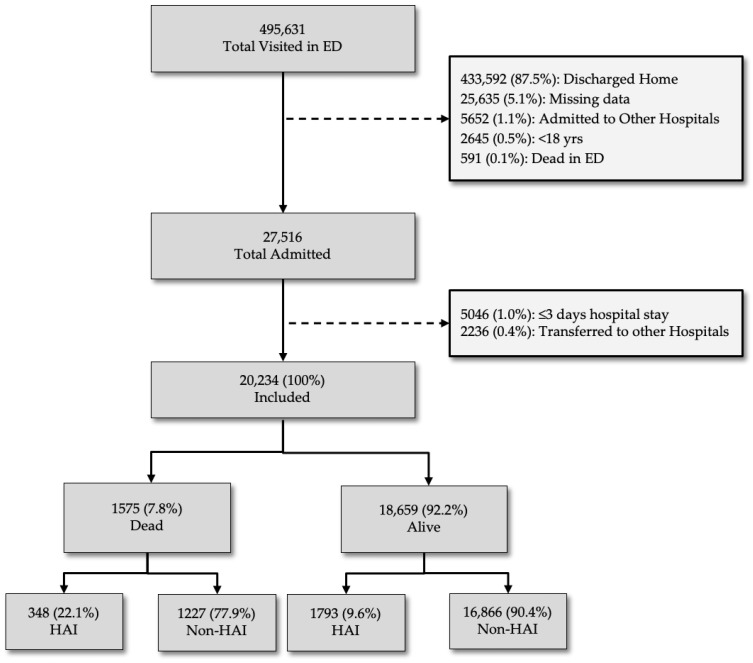
Flow diagram of the study.

Inclusion criteria required the enrolment of 20,234 subjects admitted to acute care hospital from the ED with information from both databases (Figure 1).

A total of 468,115 patients were excluded from the analysis for the following reasons: 433,592 (87.5%) were excluded because they were discharged home directly from the ED; 25,635 (5.1%) were excluded due to insufficient data quality; 2645 (0.5%) were aged <18 years, and 591 (0.1%) died in the ED. A further 5046 patients were excluded because their hospital stay was ≤3 days, and 2236 (0.4%) were excluded due to transfer to external private hospitals. The final analysis was performed on the digital health records of 20,234 patients admitted to an acute care hospital from an ED.

### 2.3. Variables

Patient characteristics (age and sex), priority levels, and vital signs were recorded at arrival in the ED. Vital signs, such as systolic blood pressure (SBP), heart rate (HR), respiratory rate (RR), and temperature, were considered in order to calculate the National Early Warning Score (NEWS), which is considered a categorical variable (0–4: low risk; 5–6: medium risk; >6: high risk) [8].

The chief complaints on arrival at the ED were assigned using the Canadian Emergency Department Information System (CEDIS) Complaints List (V2.0) classification [9]. All presenting complaints were further divided into two categories: traumatic vs. non-traumatic origin.

The Charlson Comorbidity Index (CCI) [10] was calculated using information from the free text fields of the digital health records. The CCI (mild, 1–2; moderate, 3–4; severe, >4) was calculated using recently validated selection criteria and disease categories [11]. Age adjustments were applied, with 1 point added for each decade over 40 years of age (e.g., 50–59 years, +1; 60–69 years, +2; 70–79 years, +3, etc.), with these “age points” added to the total CCI score [10].

Healthcare-acquired infections were classified according to ECDC disease categories [4,5] and were as follows: pneumonia (PN), urinary tract (UT) infections, bloodstream (BS) infections, gastrointestinal (GI) infections, surgical site (SS) infections, and skin and soft tissue (SST) infections.

Subjects with a diagnosis of HAI were those who had (a) at least one of the diagnoses included in one of the six ECDC disease categories [4]; (b) hospital stay >3 days [4]; (c) no diagnosis of infection at the time of hospital admission; (d) diagnosis of HAI during the hospital stay only as the 3rd–6th diagnosis in subjects with a 1st or 2nd diagnosis of non-infectious disease; (e) confirmatory information from clinical, laboratory, microbiological and radiological electronic health records. If a patient had more than one HAI, they were counted only once.

In the statistical model, we also tested the following variables: EDLoS (hours), overnight stay in the ED between midnight and 8 a.m., and weekend vs. work-day.

### 2.4. Statistical Analysis

The data were summarised as counts and percentages. Continuous variables were reported as either the mean (standard deviation, SD) or median [interquartile range, IQR]. Differences in patient characteristics, along with the corresponding 95% confidence intervals (CIs), were calculated using the Agresti–Caffo method [11]. For all statistical analyses, we set the significance level at *p* < 0.001.

The primary outcome measure was 30-day mortality following an ED visit. The demographic characteristics taken into consideration were age, sex, and comorbidities.

Comorbidities were considered both as individual variables and as a categorised value of CCI > 4. Additional variables tested were NEWS, priority levels (0 to 4) [12], ICD9-CM main diagnosis codes, trauma and non-trauma related visits, ED length of stay (EDLoS), calculated as the difference between entry and exit times as a categorical value (<6, 7–12, 13–24, >24 h), in-hospital length of stay (iHLoS) (days), overnight stay (from midnight to 08:00 a.m.), and week-end vs. week-day.

A Cox proportional hazard model was used for estimating the association between HAI status and mortality risk. The odds ratio (OR) and 95% confidence intervals (95% CIs) were calculated. A score for mortality risk was calculated for each patient based on the coefficients computed by the logistic model derived from variables entering the stepwise procedure.

The accuracy of such a risk score was evaluated by the area under the receiver operating characteristic (ROC) curve. The optimal cutoff point was calculated by the Youden index [13]. To avoid ambiguity, no synthetic data were generated to address missing data; hence, the analysis utilised only complete cases. For feature selection, stepwise feature selection is used [14].

Using a two-tailed binomial test, we estimated that, for a study with adequate statistical power and an acceptable margin of error of ±1%, assuming a significance level (α) of 5%, a power of 80%, and an expected prevalence of HAI cases of 7.7%, as reported in the ECDC’s last publication [4], the required initial sample size would be at least 2731 patients, a lower number of cases than what was considered. The logistic model used a rule of thumb of at least 10 events per variable. A model with 10 to 20 predictors would require at least 200 cases of HAI. Assuming an incidence rate of 7.7%, this would be about 2600 patients, in line with the previous estimate. Statistical analyses were conducted in Python (version 3.10.12), with the libraries lifelines (version 0.30.0), sklearn (version 1.6.1) and SciPy (version 1.16.3).

## 3. Results

### 3.1. Participants

Out 27,516 hospitalised patients, 20,234 were included in our cohort and considered for the analyses. A total of 5046 cases (18.3%) were excluded because their hospital stay was ≤3 days and 2236 (8.1%) due to being transferred to different hospital facilities (Figure 1).

### 3.2. Descriptive Data

The characteristics of patients in the two groups are summarised in Table 1. No differences were found between men and women, and the age was 79 (20) years (median(IQR)), but when the different age groups were compared, patients over 80 were more likely to be in the deceased group. Patients with a high-risk NEWS and those with a comorbidity index (CCI) greater than 4 were also more likely to be in the deceased group.

The most reported comorbidities in order of frequency were DM, HF, D COPD, CAD and CKD, Table S1 (Supplementary). All of these conditions were more prevalent in the deceased group.

The median length of EDLoS was 7 (10)hours, median (IQR), with 40.1% of cases lasting less than 6 h, 16.2% lasting between 12 and 24 h, and 13.5% lasting more than 24 h.

The main diagnoses by category in order of frequency according to the ICD-9-CM classification system are listed in Table S2 (Supplementary). The most frequently reported diagnoses were those of respiratory diseases (21.7%) and circulatory diseases (20.5%). When the deceased vs. alive groups were compared, respiratory, infectious and parasitic disease and neoplasm diagnoses were more represented in the dead group, while all other diseases were more represented in the alive group.

HAI diagnoses in order of frequency, divided by category according to ECDC criteria, are reported in Table 2. The majority (90%) of these diagnoses belonged to the pneumonia (PN), urinary tract infection (UTI) and bloodstream infection (BS) categories.

### 3.3. Outcome Data

A total of 1575 out of 20,234 cases (7.8%) admitted to the hospital died during their stay. The percentage of subjects with HAIs was higher in the group of deceased patients (22.1%; 95% CI 20.1–24.2) than in subjects discharged alive (9.6%; 95% CI 9.2–10.0); *p*-value < 0.001 (Figure 1).

### 3.4. Main Results

**Figure 2:**
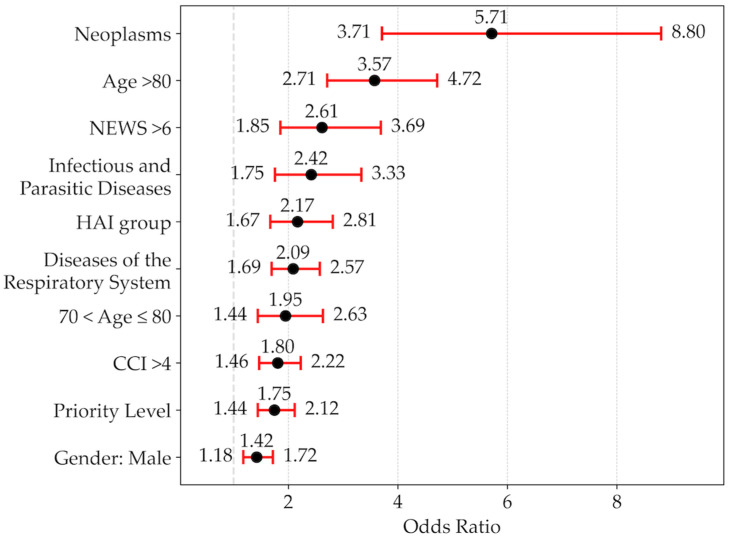
Forest plot of the selected variables entered into the Cox hazard model in the prediction of mortality. Data are reported in order of importance as hazard ratios and 95% confidence intervals.

The list of independent variables selected from the Cox hazard model to predict 30-day mortality is shown in order of importance in Figure 2. Among those selected from the model, one of the most important was a diagnosis of HAI.

Variables not included in the model were individual comorbidities, EDLoS, overnight stay in the ED, trauma-related visits, diagnoses of neoplasms (140–239), endocrine, nutritional, metabolic diseases and immune system disorders (240–279), diseases of the blood and blood-forming organs (280–289), diseases of the nervous system and sense organs (320–389), mental and behavioural disorders (290–319), digestive system diseases (520–579), genitourinary system diseases (580–629), musculoskeletal system and connective tissue diseases (710–739), complications of pregnancy, childbirth, and puerperium (630–679), symptoms, signs, and laboratory findings (780–799), injury and poisoning, external causes (800–999), and external causes (E, V codes).

Figure S1 (Supplementary) shows the ROC curve for the mortality risk score, which was calculated using the coefficients from the logistic regression. The model’s accuracy in predicting the outcome was 0.804 ± 0.012, with a sensitivity of 84% and a specificity of 65% at the optimal cutoff point (score of 0.470), achieving maximum sensitivity and specificity simultaneously.

**Figure 3:**
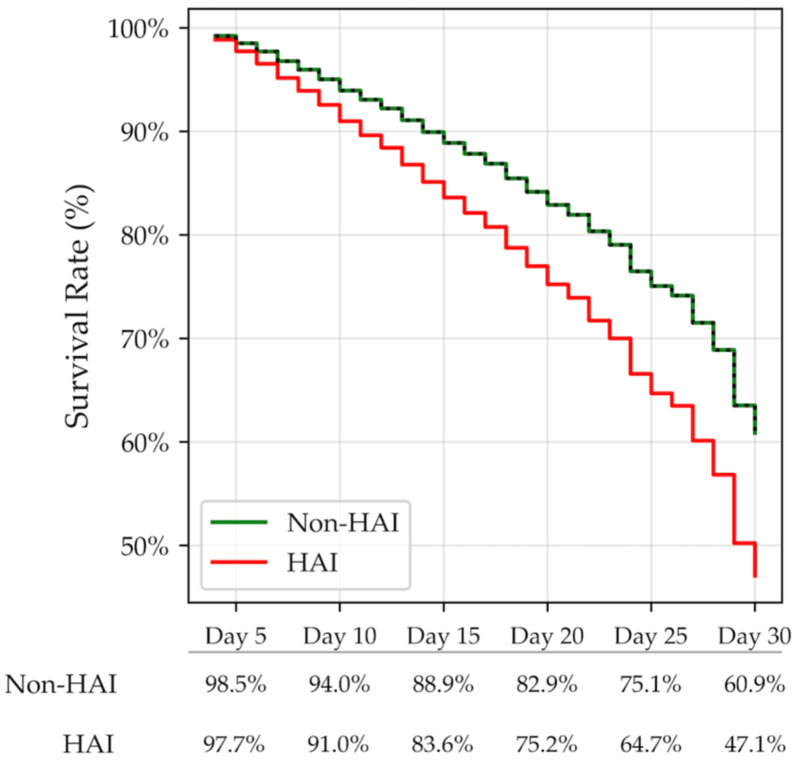
Cumulative survival rate for the 2 different groups, HAI (red line) vs. non-HAI category (green line). The log-rank test (Mantel–Cox test) was used to compare the survival distributions (%) between the different groups.

The 30-day survival rate was lower for the HAI group (47.1%) than for the non-HAI group (60.9%) (Figure 3), with an increased risk for the HAI group (hazard ratio 1.518; 95% CI: 1.338–1.721; *p*-value: <0.001).

Subsequent analysis, which divided HAIs into individual categories, revealed that the percentage of 30-day survivors was particularly low among subjects with BS and PN (left), with a particularly high mortality hazard ratio shown on the right side of the figure (see Figure S2 in the Supplementary Materials).

## 4. Discussion

### 4.1. Key Results

This retrospective, multicentre study found that HAIs were associated with an increased risk of mortality after adjusting for all other predictive variables selected by the statistical model. This risk was particularly elevated for patients who had contracted infections such as pneumonia or bloodstream infections.

Our study protocol considered cases with HAI, selecting them on the basis of criteria specified by the ECDC [4], with the effort to minimise false positives, i.e., cases with an infectious disease already present at the time of admission, but with symptoms not yet evident at the time of admission [15]. The percentage of these cases could be problematic: the ECDC’s latest report estimates that these cases range from 15.6% (Czech Republic) to 41.2% (Sweden) [16]. We believe that our decision did not introduce significant selection bias [17]. Nonetheless, it should be noted that it would be very difficult to establish reliable criteria for determining the exact date of diagnosis anyway, given the numerous variables to consider.

In our analysis, we used ICD-9-CM diagnostic codes to identify nosocomial infections. Past studies indicate that coded diagnoses had high specificity (≥93%), particularly for SS, Clostridium and BS infections [18]. In our study, these cases accounted for 8.9%, which is in line with the official ECDC registries (7–10% for the Italian registry). Retrospective analysis of cases may lead to overestimation, but we believe that the case selection criteria minimised the risk of false positives. However, as this is a retrospective study, this risk of bias in sample selection must unfortunately be accepted. Finally, it should be noted that the patient’s medical history could not be complete due to regulatory and technical constraints that prevented access to data from other institutions. However, the application of a multivariate regression model that corrects the results for confounding variables should have contained any initial errors [19].

The incidence of healthcare-associated infections has been reported to be associated with risk factors such as advanced age and male gender. The site of infection is also important. It should also be noted that infection rates could vary in different geographical areas, hospital settings and levels of care. For instance, infections are more prevalent in intensive care and specialist areas than in general wards. In EDs, the risk mainly arises from overcrowding, the complexity and severity of diseases, and invasive procedures being performed in an unprotected environment. In this setting, respiratory infections (such as pneumonia), bloodstream infections and urinary tract infections appear to be the most common [20]. In our dataset, a diagnosis of HAI was recorded in 10.6% of patients admitted from the emergency department, a figure consistent with the official data reported in the European registry [4]. It should be noted that this percentage was higher in deceased patients (22.1%) than in survivors (9.6%) and that the most frequent nosocomial infections were, in order, PN (41%), UTI (34.3%) and BS (26.5%), although in the proportional risk model only BS and PN were associated with the risk of death (Figure S2).

A primary cancer diagnosis was one of the main risk factors associated with mortality. It is well known that a significant percentage of patients receive their first cancer diagnosis during an emergency room visit [21] and that these cases, often immunocompromised, have an increased risk of infection and death [22]. The types of cancer with the highest risk of infection and death are those of the pancreas, lung, biliary tract, anaplastic thyroid, and head and neck [22]. In our series, there were 1135 cases of primary cancer, accounting for 4.5% of all cases. Among these, the most common were pancreatic (1 in 7 cases), lung (1 in 6) and colon (1 in 5) cancer.

In patients with HAI, advanced age (namely in the over-80 age group) and male gender have been identified as risk factors for mortality [23]. Our analysis confirms that advanced age in both the 70–80 and >80 age groups, as well as male gender, are associated with an increased risk of death. The inclusion of only the most advanced age categories in the model may be explained by the stepwise procedure used in the logistic model, which prioritises the most important variables.

The National Early Warning Score (NEWS) is a clinical tool currently used in EDs to estimate the risk of adverse outcomes and short-term mortality. Its reliability appears to be particularly high in predicting the risk of adverse events in elderly patients (>65 years) admitted to hospital for acute care, particularly in elderly patients with pneumonia [24]. In our series, only high-risk NEWS, present in 6.7% of cases, was confirmed among the variables included in the logistic model.

The logistic model included infectious or parasitic diseases and respiratory diseases among the group of main diagnoses associated with an increased mortality risk (Figure 2). Patients with these diagnoses are known to be at high risk of developing further infections if they are immunocompromised [4] or if they spend long periods of time in crowded environments such as the ED [15]. In our series, the most frequent primary diagnoses in the infectious diseases category were E. Coli sepsis, unspecified sepsis and Staphylococcus aureus sepsis. In the respiratory system diseases category, the most frequent primary diagnoses were bacterial pneumonia, COPD with exacerbation and pneumonia caused by SARS-CoV2.

It should be noted that, in some cases, HAIs belonging to the same category as the primary diagnosis developed; for example, PN developed in patients with a primary diagnosis belonging to the respiratory disease category. Specifically, 16.3% of cases of respiratory disease developed a PN-type HAI; 5.1% of cases with a primary diagnosis of an infectious disease developed BS; and finally, 3.0% of cases of genitourinary disease developed an additional UT infection.

A high burden of comorbidity is one of the characteristics associated with an increased risk of mortality. The CCI has been confirmed as an indicator for patient risk stratification and the prediction of adverse outcomes in hospitalised patients, particularly those with a high index. In our study, the CCI was high (≥4) in 47.8% of cases, with a higher prevalence (75.4%) among deceased patients than survivors. Diabetes is a particular risk factor for infection, especially in cases of poor glycaemic control and in elderly patients [7]. In our study, the most common comorbidities were, in order of frequency, ST, D, COPD, DM and CKD. All of these were more prevalent in the deceased group than in the survivors.

### 4.2. Limitations

Several limitations of this study must be acknowledged: First, the data were collected from multicentre first-level EDs of the same healthcare area. These results were obtained from an area with a uniform healthcare level: it may be that our results would not be confirmed if the level or organisation of health services differed from that in the study area.

Second, we used ICD-9-CM diagnosis codes to identify HAIs. Retrospective analysis of cases could generate a risk of case selection bias, but the selection criteria we used should minimise the risk of false positives. However, as this is a retrospective study, this risk must unfortunately be accepted and cannot be eliminated.

Third, the accuracy of the logistic model in predicting the event was 0.804 [SE 0.012], with an optimal cutoff point of 0.417, a level of discriminatory power considered acceptable for clinical purposes. Unfortunately, it is not possible to understand whether the group of selected variables has such a high impact on the accuracy of the patient’s medium- and long-term prognosis. To achieve this result, it would be essential to minimise the risk of distortion through careful selection of the variables to be tested, good outcome measures, and careful analysis of the results, eliminating the so-called self-fulfilling prophecy effect. Additional clinical events, such as complications and severity scores, with appropriate use of statistical models (e.g., survival analysis including time to event or Bayesian models), could improve predictive performance [18].

### 4.3. Generalizability

Although the sample analysed was large, the data were collected from four first-level EDs in an area with a homogeneous healthcare model. Therefore, we cannot rule out the possibility that the results may not be fully replicable in areas with different organisational models.

## 5. Conclusions

This large, multicentre study concludes that a series of variables available at the time of ED admission can predict the risk of developing HAIs and, therefore, the risk of death during the hospital stay. The risk is particularly high for patients who contract pneumonia or bloodstream infections. Future studies will need to assess whether providing additional information on patient characteristics, diagnoses and treatment pathways could reduce the risk of complications, particularly HAIs, and thereby improve outcomes.

## Figures and Tables

**Table 1:** Baseline characteristics of the 2 groups in relation to the presenting clinical profile.

	Total, No.	Dead, No. (%)	Alive, No. (%)	OR (95% CI)	*p*-Value
Patients	28,803	2359 (9.3)	22,930 (90.7)	--	--
**Sex** (males)	12,694 (50.2)	1177 (50.1)	11,517 (50.2)	0.99 (0.91–1.08)	0.896
**Age** (years)	78 [64–86]	86 [79–91]	77 [18–106]	--	NS
18–30	998 (3.9)	3 (0.1)	995 (4.3)	0.03 (0.01–0.09)	<0.001
31–40	885 (3.5)	8 (0.3)	877 (3.8)	0.09 (0.04–0.17)	<0.001
41–50	1274 (5.0)	19 (0.8)	1255 (5.5)	0.14 (0.09–0.22)	<0.001
51–60	2238 (8.8)	64 (2.7)	2174 (9.5)	0.27 (0.21–0.34)	<0.001
61–70	3256 (12.9)	175 (7.4)	3081 (13.4)	0.52 (0.44–0.61)	<0.001
71–80	5695 (22.5)	418 (17.8)	5277 (23.0)	0.72 (0.65–0.81)	<0.001
>80	10,934 (43.2)	1663 (70.8)	9271 (40.4)	3.57 (3.25–3.91)	<0.001
**NEWS**					
0–3	21,317 (84.3)	1488 (63.3)	19,829 (86.5)	0.27 (0.25–0.30)	<0.001
4–6	2258 (8.9)	406 (17.3)	1852 (8.1)	2.38 (2.11–2.67)	<0.001
>6	1705 (6.7)	456 (19.4)	1249 (5.4)	4.18 (3.72–4.70)	<0.001
**CCI**					
1–2	5563 (22.1)	66 (2.81)	5497 (24.0)	0.09 (0.07–0.12)	<0.001
3–4	7632 (30.2)	512 (21.8)	7120 (31.0)	0.62 (0.56–0.68)	<0.001
>4	12,085 (47.8)	1772 (75.4)	10,313 (45.0)	3.75 (3.40–4.13)	<0.001

Data presented as number of cases (N), and percentages (%), with odds ratios (OR) and 95% confidence intervals (95% CI). Statistical significance se at *p*-value of <0.001. NEWS: New Early Warning Score, CCI: Charlson Comorbidity Index.

**Table 2:** Healthcare-associated infections (HAIs) by category. Data are listed, in order of frequency, as the number of cases (No.) and percentages (%).

		No. = 2141	%
**Pneumonia (PN)**			
485	Bronchopneumonia, unspecified	217	10.1
460–466	Acute and lower respiratory tract infections	115	5.4
486	Pneumonia, unspecified	162	7.6
480	Viral pneumonia	44	0.2
482–3, 484.0–7–8, 487–8	Pneumonia due to another organism	352	16.4
481	Pneumococcal pneumonia	29	0.1
	Total	890	41.6
Urinary tract (UT)			
599.0	Urinary infection (unspecified site)	697	32.5
590.1	Acute pyelonephritis	24	0.1
996.64	Infection due to a urinary catheter	13	0.1
	Total	734	34.3
**Bloodstream (BS)**			
995.91, 995.92	Sepsis	181	8.4
038, 038.4	Septicaemia (including specific organisms)	149	7.0
038.8, 038.9, 790.7	Septicaemia, unspecified; bacteriemia	139	5.6
038.0–1	Septicaemia due to Streptococcus	98	4.6
038.2	Septicaemia due to Gram-neg. bacteria	1	0.0
	Total	568	26.5
**Gastrointestinal (GI)**			
008.45	Intestinal infection (Clostridium difficile)	76	3.5
009.0–3	Infectious enteritis, unspecified	33	1.5
008.5x, 009.2, 099.3	Other intestinal infections due to bacteria	5	0.1
	Total	114	5.3
**Surgical Site (SS)**			
996.6x	Infection due to an internal prosthetic device	33	1.5
998.5–9	Other post-operative infection	2	0.1
998.50–4, 998.51	Postoperative wound infection,	12	0.1
	Total	47	2.1
**Skin and Soft Tissue (SST)**			
680–686	Other cellulitis and abscess	40	0.2

## Data Availability

The current study’s generated/analysed datasets are available upon request.

## Supplementary Materials

The following supporting information can be downloaded at https://www.mdpi.com/article/10.3390/jcm15041483/s1, Table S1. Comorbidities in subjects attending the ED. Data presented as number of cases (%), with Odds Ratio (OR) and 95% Confidence Intervals (95% CI). Statistical significance set at *p* < 0.001. Table S2. Main diagnosis codes, reported as the number of cases (%) and Odds Ratio (OR) with 95% confidence intervals (95% CI). Statistical significance set at < 0.001. Figure S1. ROC plots of the risk score obtained from the logistic model, evaluating its ability to identify the increased mortality risk. Figure S2. Survival rate with per cent of 30-day survival rate (left panel) and hazard ratio with 95% confidence intervals (right panel) in relation to individual HAI categories. SST, skin and soft tissue; SS, surgical site; UT, urinary tract; GI, gastrointestinal; PN, pneumonia; BS, bloodstream.

## Abbreviations

The following abbreviations are used in this manuscript:

BS	Bloodstream
CCI	Charlson Comorbidity Index
CHF	Congestive Heart Failure
HM	History of Hemiplegia
CKD	Chronic Kidney Disease
COPD	Chronic Obstructive Pulmonary Disease
CVA	Acute Cerebrovascular Accidents or Transient Ischemic Attacks
D	Dementia
DM	Diabetes Mellitus
ED	Emergency Department
EDLos	Emergency Department Length of Stay
HLoS	In-Hospital Length of Stay
GI	Gastrointestinal
GTD	Connective Tissue Disease
PUD	Peptic Ulcer Disease
HAI	Healthcare-Associated Infection
HR	Hazard Ratio
L	Lymphoma and Leukaemia
LD	Liver Disease
MI	History of Myocardial Infarction
NEWS	National Early Warning Score
PN	Pneumonia
PVD	Peripheral Vascular Disease
ROC	Receiver Operating Characteristic
SS	Surgical Site
SST	Skin and Soft Tissue
ST	Solid Tumours
UTI	Urinary Tract
