# Supplementary material for: Healthcare-Associated Infections Impact Mortality in Patients Admitted to the Acute Care Hospital from the Emergency Department
Source: J Clin Med. 2026 Feb 13;15(4):1483. doi: 10.3390/jcm15041483 (PMC12942601; doi:10.3390/jcm15041483)
Supplement: Supplementary file 1 [file jcm-15-01483-s001.zip › Supplementary Table S1.pdf]

Table S1.

Comorbidities	Total, No. (%)	Dead, No. (%)	Alive, No. (%)	OR (95% CI)	P-value
**Sex (males)**	**12,694 (50.2)**	**1,177 (50.1)**	**11,517 (50.2)**	**0.99 (0.91–1.08)**	**0.896**
D	4,157 (16.4)	459 (19.5)	3,698 (16.1)	1.26 (1.13–1.41)	<0.001
COPD	3,657 (14.5)	534 (22.7)	3,123 (13.6)	1.86 (1.68–2.07)	<0.001
DM	3,371 (13.3)	671 (28.5)	2,700 (11.8)	2.99 (2.71–3.30)	<0.001
CKD	2,678 (10.6)	457 (19.4)	2,221 (9.7)	2.25 (2.01–2.52)	<0.001
CVA	2,669 (10.6)	345 (14.7)	2,324 (101)	1.53 (1.35–1.72)	<0.001
MI	2,168 (8.6)	218 (9.3)	1,950 (8.5)	1.10 (0.95–1.27)	0.203
CHF	2,166 (8.6)	344 (14.6)	1,822 (7.9)	1.99 (1.76–2.25)	<0.001
HM	1,881 (7.4)	257 (10.9)	1,624 (7.1)	1.61 (1.40–1.85)	<0.001
LD	1,656 (6.5)	203 (6.8)	1,453 (6.3)	1.40 (1.20–1.63)	<0.001
CTD	1,545 (6.1)	153 (6.5)	1,392 (6.1)	1.08 (0.91–1.28)	0.396
PVD	517 (2.0)	62 (2.6)	455 (2.0)	1.34 (1.02–1.75)	0.033
PUD	470 (1.91)	52 (2.2)	418 (1.8)	1.22 (0.91–1.63)	0.183
L	406 (1.6)	63 (2.7)	343 (1.5)	1.81 (1.38–2.38)	<0.001
Ly	142 (0.6)	20 (0.8)	1 22 (0.5)	1.60 (1.00–2.58)	0.001
AIDS	13 (0.1)	0 (0.0)	13 (0.6)	NA	NA

Abbreviations: diabetes mellitus (DM), solid tumors (ST), dementia (D), chronic obstructive pulmonary disease (COPD), acute cerebrovascular accidents or transient ischemic attacks (CVA), chronic kidney disease (CKD), history of myocardial infarction (MI), congestive heart failure (CHF), hemiplegia (HM), liver disease (LD), connective tissue diseases (CTD), peripheral vascular disease (PVD), peptic ulcer disease (PUD), leukemia (L), lymphoma (Ly), Acquired Immune Deficiency Syndrome (AIDS).
