# Supplementary material for: Healthcare-Associated Infections Impact Mortality in Patients Admitted to the Acute Care Hospital from the Emergency Department
Source: J Clin Med. 2026 Feb 13;15(4):1483. doi: 10.3390/jcm15041483 (PMC12942601; doi:10.3390/jcm15041483)
Supplement: Supplementary file 1 [file jcm-15-01483-s001.zip › Supplementary Table S2.pdf]

Table S2.

**ICD-9 Diagnosis codes**	**Total, No.**	**Dead, No. (%)**	**Alive, No. (%)**	**OR (95% CI)**	**p-value**
Diseases of the Respiratory System 460-519)	5,478 (21.7)	910 (38.7)	4,568 (19.9)	2.54 (2.32–2.78)	<0.001
Diseases of the Circulatory System (390-459)	5,195 (20.5)	395 (16.8)	4,800 (20.9)	0.76 (0.68–0.85)	<0.001
Diseases of the Digestive System (520-579)	3,343 (13.2)	171 (7.3)	3,172 (13.8)	0.49 (0.42–0.57)	<0.001
Injury and Poisoning (800-999)	2,605 (10.3)	65 (2.8)	2,540 (11.1)	0.23 (0.18–0.29)	<0.001
Diseases of the Genitourinary System (580-629)	2,026 (8.0)	139 (5.9)	1,887 (8.2)	0.70 (0.59–0.84)	<0.001
Infectious and Parasitic Diseases (001-139)	1,926 (7.6)	348 (14.8)	1,578 (6.9)	2.35 (2.08–2.66)	<0.001
Neoplasms (140-239)	1,135 (4.5)	185 (7.9)	950 (4.1)	1.98 (1.68–2.33)	<0.001
Symptoms, Signs, & Lab. Findings (780 – 799)	981 (3.9)	50 (2.1)	931 (4.1)	0.51 (0.39–0.68)	<0.001
Mental Disorders (290-319)	623 (2.5)	3 (0.1)	620 (2.7)	0.05 (0.01–0.14)	<0.001
Diseases of the Nervous System (320-389)	490 (1.9)	26 (1.1)	464 (2.0)	0.54 (0.36–0.81)	0.002
Diseases of the Blood (280-289)	483 (1.9)	18 (0.8)	465 (2.0)	0.37 (0.23–0.60)	<0.001
Endocrine, Nutritional, and Metabolic (240-279)	339 (1.3)	24 (1.0)	315 (1.4)	0.74 (0.49–1.12)	0.157
Diseases of the Musculoskeletal System (710-739)	230 (0.9)	11 (0.5)	219 (1.0)	0.49 (0.27–0.89)	0.018
Complications of Pregnancy (630-679)	224 (0.9)	0 (0.0)	224 (1.0)	NA	NA
Diseases of the Skin (680-709)	112 (0.4)	4 (0.2)	108 (0.5)	0.36 (0.13–0.98)	0.037
External Causes (E, V codes)	70 (0.3)	1 (0.1)	69 (0.3)	0.14 (0.02–1.02)	0.023
Congenital Malformations (740-759)	20 (0.1)	0 (0.0)	20 (0.1)	NA	NA
