# Supplementary figures and images for: Healthcare-Associated Infections Impact Mortality in Patients Admitted to the Acute Care Hospital from the Emergency Department
Source: J Clin Med. 2026 Feb 13;15(4):1483. doi: 10.3390/jcm15041483 (PMC12942601; doi:10.3390/jcm15041483)

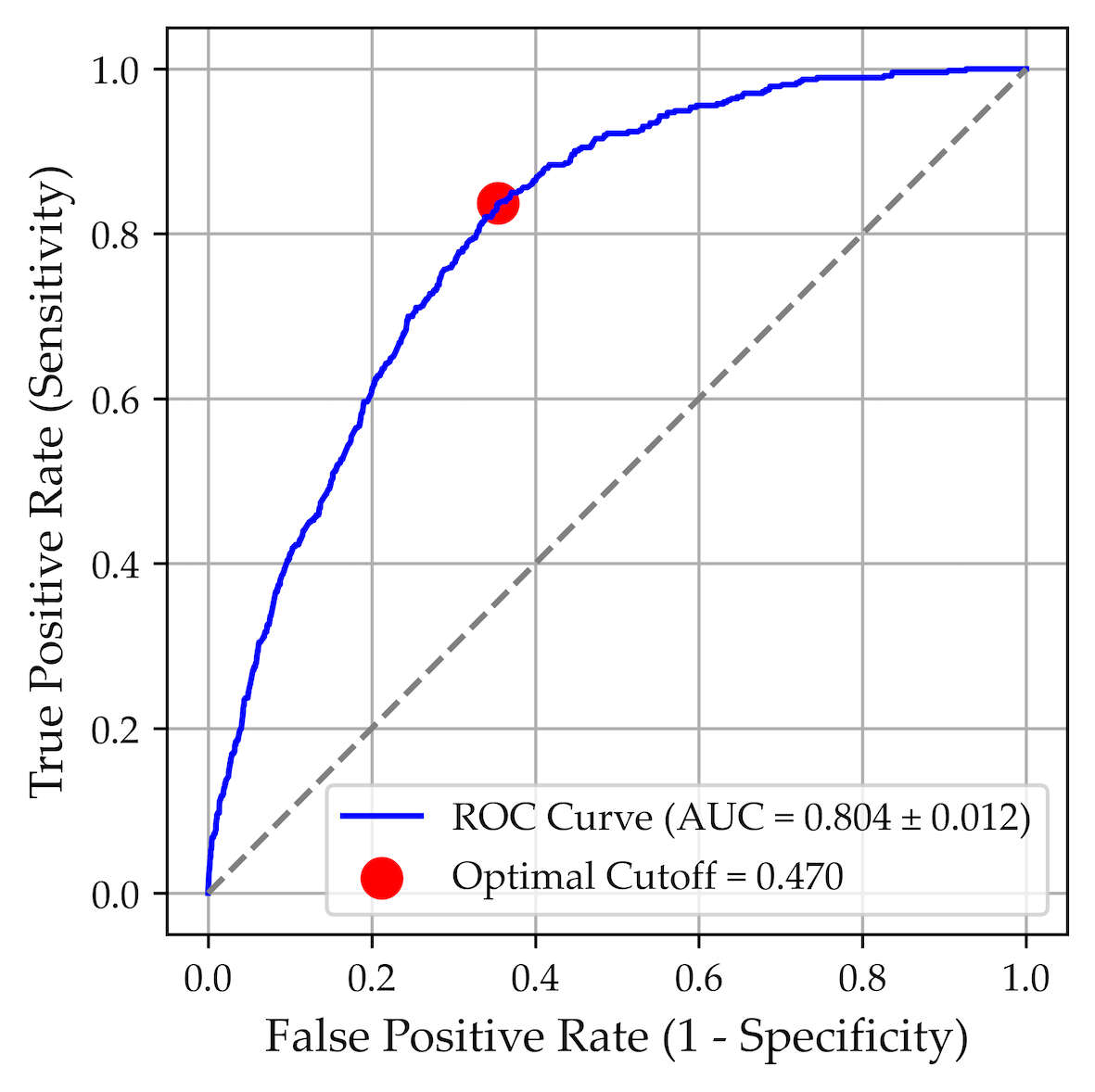

Supplement: Supplementary file 1 [file jcm-15-01483-s001.zip › Supplementary Figure S1.tiff]

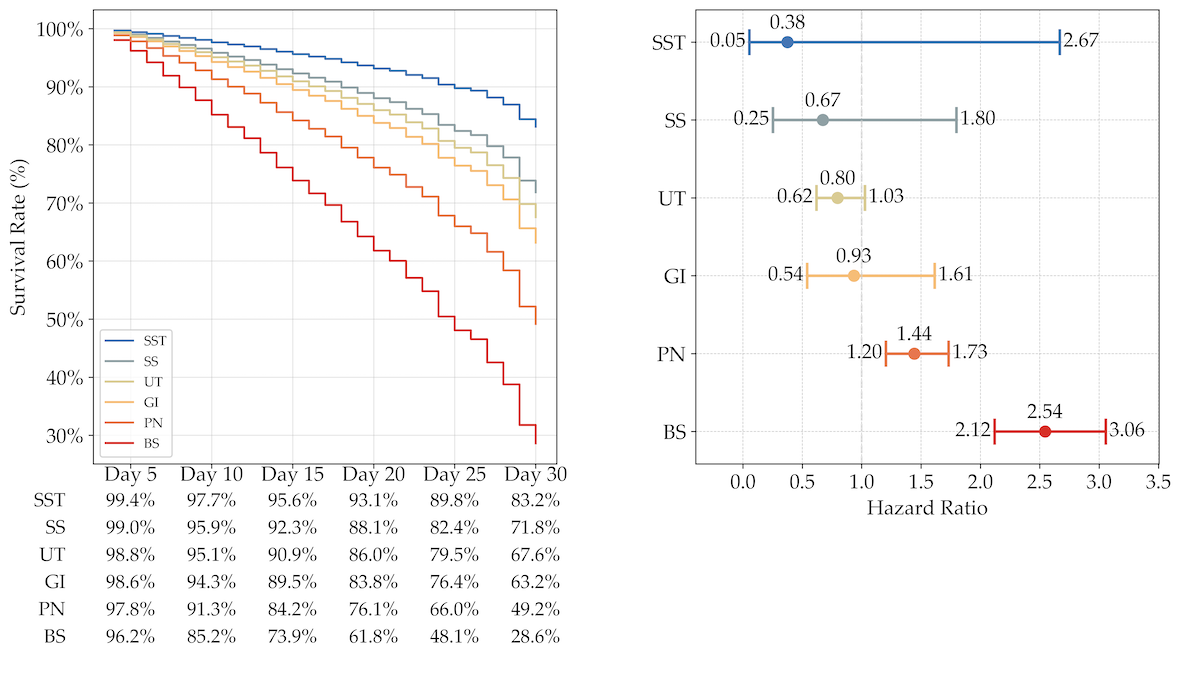

Supplement: Supplementary file 1 [file jcm-15-01483-s001.zip › Supplementary Figure S2.tiff]
